# Deciphering the genetic alterations in matrix metallo-proteinase gene family and its putative association with head and neck squamous cell carcinoma

**DOI:** 10.22099/mbrc.2020.38344.1544

**Published:** 2021-03

**Authors:** Jambukeswaran Aparna, Aseervatham Selvi Smiline-Girija, Arumugam Paramasivam, Jayaseelan Vijayashree-Priyadharsini

**Affiliations:** 1 Department of Microbiology, Saveetha Dental College, Saveetha Institute of Medical and Technical Sciences (SIMATS), Saveetha University, Chennai 600077, India; 2 Biomedical Research Unit and Laboratory Animal Centre-Dental Research Cell, Saveetha Dental College, Saveetha Institute of Medical and Technical Sciences (SIMATS), Saveetha University, Chennai 600077, India

**Keywords:** Matrix metalloproteinases, head and neck squamous cell carcinoma, survival

## Abstract

Matrix metallo-proteinases (MMPs) a group of zinc-dependent proteolytic enzymes which play a key role in tumorigenesis by degrading almost all extracellular matrix (ECM) components. MMPs are associated with tumour progression including invasion, angiogenesis, metastasis and poor prognosis. Genetic alterations such as single nucleotide variations and other gross chromosomal abnormalities have been found to drive the process of malignant transformation. In line with the above facts, the present study aims to analyse the genetic alterations, associated gene expression patterns and survival probability of HNSCC patients upon differential expression of the crucial members of the MMP family. The observational study utilised several computational tools. The cBioportal database was used as the primary source of identification of genetic alterations in the MMP family of genes. The Cancer Gene Atlas dataset (Firehose Legacy) was used for the investigations. The highest frequency of alteration was identified in the *MMP20* gene (8%). The common gene alterations were amplifications, deep deletions, mis-sense and truncating mutations. Interestingly, amplification and deep deletion followed the same pattern in about 31 patients, in genes MMP1, 3, 7, 8, 10, 12, 20, and 27. The *MMP20* gene expression analysis showed a significant difference between the normal subjects and the patients with primary tumors (6.95 x 10^-4^). The Kaplan-Meier survival curve analysis identified that female patients with high-level expression of the *MMP20* gene had a low survival probability when compared to male HNSC patients. Taken together, the present study provides preliminary information about the involvement of the *MMP20* gene of the MMP family with HNSCC. Further experimental analysis is required to derive a strong association between the gene alterations observed with HNSCC.

## INTRODUCTION

Head and neck squamous cell carcinoma is the most common form of cancer with a mortality rate of 40-50%. HNSCC stands sixth among the cancer types [1], with an increased incidence clustered in certain geographical regions such as the south Asian countries including India, Pakistan and Srilanka [2]. The risk factors associated with the disease are smoking and usage of smokeless tobacco such as pan, gutka etc., alcoholism, infections with human papilloma virus (HPV), poor oral hygiene, sharp tooth and other environmental factors. Several genetic markers have been identified to be associated with the process of tumorigenesis in HNSCC. In recent years studies on extracellular matrix proteins have gained interest due to their involvement in cell growth, survival, differentiation and motility. The local degradation of ECM enables tumor cells to metastasize from the tumor site to other organs. Matrix metalloproteinases are enzymes which are responsible for the breakdown of ECM. The MMP family consist of 23 genes, which have been implicated in the tumor invasion and progression [3]. They belong to zinc dependent ECM remodelling endopeptidase possessing the ability to degrade components of ECM [4]. The gene expression of MMPs is regulated by growth factors, stress, oncogenic transformations and cell-cell interactions. The responsive members of the MMP family of genes contain activator protein-1 (AP-1) binding site at the proximal promoter site. The proto-oncogene *Jun *and *Fos *bind to the AP-1 element which in tun activates the transcription of the *MMP *gene [5,6].

A study by Nandha et al, demonstrated that regulation of matrix metalloproteinases of types MMP2 and MMP9 could aid in understanding the invasive potential of tumors. Clinico-pathology, histopathology and TNM grading were performed on tumor and adjacent normal tissues. Zymography, immuno-histochemistry, ELISA, western-blot and real time PCR were used to assess the enzyme activity. A differential expression pattern of several genes were observed. An increased level of MMP9 was accompanied by an increase in fibronectin, protein kinase B, focal adhesion kinase, phosphatidylinositol 3-kinase and reduced expression of tissue inhibitor of metalloproteinase-1. Thus it was proved that MMP9 has a vital role to play in tumor invasion and metastasis and hence can be used as a marker to demonstrate the metastatic potential of tumors [7]. Alterations or aberrations in genes encoding proteins involved in the process of tumorigenesis could act as important biomarkers to identify the risk or susceptibility of an individual to develop HNSCC [8]. Several studies performed earlier were based on differential gene expression analysis, which revealed the association of those genes with HNSCC. The rationale of the present study was to identify genetic variants in the MMP family of genes and to deduce the possible consequences of such alterations in the development of HNSCC. In addition, the study also throws light on the differential expression profile of the gene carrying the highest frequency of alterations along with an observation on the survival probability of the altered gene.

## MATERIALS AND METHODS

The present study follows a retrospective observational study design. The source of patient’s data was procured from the cBioportal database. This database hosts an exhaustive collection of patient’s details from different cohorts (Table 1). The TCGA, Firehose legacy data set consisted of 528 head and neck squamous cell carcinoma cases of which sequencing and copy number alteration data were available for 512 tumor samples. A complete profile of mutated, amplified, deleted genes was available for each of the cases in the dataset. The demographic details of the cases in the dataset have been provided in Table 1. A list of vital genes related to the MMP family was obtained from the "HUGO Gene Nomenclature Committee at the European Bioinformatics Institute" (www.genenames.org/data/) database [9]. User defined queries based on these genes were submitted in the cBioportal database and the resultant Oncoprint data was used for further analysis [10, 11].

**Oncoprint data analysis** **:** The Oncoprint data provides information on the frequency distribution of variations in each of the genes selected, type of variation, changes in the protein coding amino acids, gene amplification, deletions, insertions, frameshifts, splice site mutations etc. These details can be used to (a) derive a putative association between the disease phenotype and genotype, (b) identify the variations in less understood pathways or genes, and (c) identify any novel variations which can be associated with the disease phenotype [10, 11].

**Table 1:** Demographic details of patients analysed in the present study

**Gender**	**Male (n = 386) Female (n = 142)**
Mutation count	6-3181
Diagnosis age	19-90 years
Smoking status	Smokers: 515 Data not available: 12 Unknown: 1
Alcohol history	Yes-352 No-165 Data not available: 11
Neoplasm Histologic grade	Grade 1: 63 Grade 2: 311 Grade 3: 125 Grade 4: 7 Grade GX:18 Data not available: 4
Race category	White: 452 African: 48 African: 48 African: 48American Indian or Alaska native: 2 Data not available: 15

**Note:** Data obtained from the cBioportal site.

**Gene expression and survival curve analysis** **:** The expression of the gene in HNSCC was analysed using the UALCAN (http://ualcan.path.uab.edu/cgi-bin/TCGA-survival1.pl?) database. Survival curve analysis based on the tumor grade and expression profile was performed to demonstrate the putative role of Rho family g genes with HNSC. Gene expression data is expressed as transcripts per million (TPM) which is a normalization method for RNA-seq data. The TPM values used for the generation of box-whisker plots were also used to determine the significant difference between the groups. The t test was performed using PERL script with the comprehensive perl archive network (CPAN) module. Combined survival effect analysis of gene expression and other clinical parameters such as race, gender, tumor grade, cancer subtypes were assessed using multivariate Kaplan-Meier survival analysis [12].

## RESULTS

Computational approach is considered to be the most widely used method by researchers because of its ease of use. An exhaustive screening could be performed using these platforms. A specific pathway or a candidate gene can be analyzed for its association with disease phenotype. The preliminary results obtained from such studies can be employed for screening of the population which could result in identification of lead molecules for the purpose of diagnosis or therapy [13, 14]. Hence in the present study the cBioportal and UALCAN database were used to assess the genetic alterations and gene expression in the HNSCC dataset. The primary database, cBioportal which hosts several datasets of which the TCGA dataset (TCGA, Firehose Legacy) was selected for the present study. The TCGA dataset consisted of 528 HNSCC patients (530 samples). The male:female ratio was found to be 2.7:1, with the diagnostic age groups ranging from 19-90 years. The number of individuals with the history of smoking and alcohol was roughly around 98% (515 individuals) and 67% (352 individuals). The dataset had samples from patients of American (85.6%), African (9.1%), Asian (2.1%) and American Indian (0.4%) descent. The distribution of patients based on the histologic grade of neoplasm is given in Table 1, of which 59% of patients had grade 2 tumor. 

**Figure 1:**
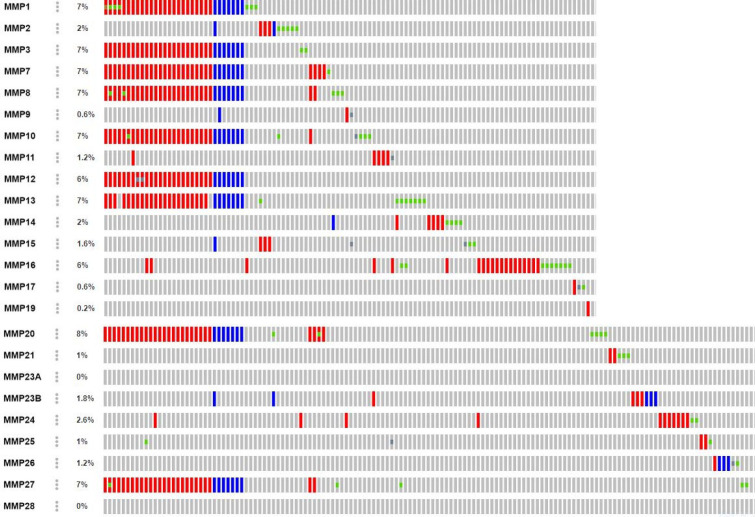
The Oncoprint data depicts the gene alterations in *MMP* family of genes. Each of the grey bar represents HNSC patients

The analysis of oncoprint data which was obtained after submission of a query for 23 genes of the MMP family demonstrated alterations ranging from 0.2 - 8%. The *MMP23A *and *MMP28 *were the only genes which did not show any kind of alterations among HNSC patients. Several non-synonymous and truncating mutations of unknown significance were identified in the present study (Table 2). Interestingly, about 31 HNSC patients presented with similar gene amplification and deep deletion in genes *MMP1, 3, 7, 8, 10, 12, 20 *and *27*. The frequency of non-synonymous mutation count was found to be high in *MMP13 *and *MMP18 *genes. Further, gene expression analysis was carried out for the gene which showed the highest frequency of gene alteration *i.e., MMP20 *[8%] (Fig. 1)*.*

**Table 2:** Details on the proteins encoded by MMP family of genes. The genetic loci, type and frequency of alterations and variant allele frequency are given in the Table

**Gene**	**Protein**	**Alteration**	**Loci**	** % of alteration**	**Variant allele frequency in tumor sample**
***MMP1***	Matrix Metallopeptidase 1 (Interstitial Collagenase)	Gene Amplification Deep deletion F308L L224W R214C L11del T451M P412H A335V	11q22.2	7	0.29 0.07 0.08 0.02 0.20 0.53 0.0051
					
***MMP2***	Collagenase Type IV-A	Gene amplification Deep deletion T377A W657R E177G S32L D142N	16q12.2	2	0.13 0.04 0.22 0.16 0.13
***MMP3***	Matrix Metalloproteinase 3 (Stromelysin 1, Progelatinase)	Gene amplification Deep deletion D124H D170A	11q22.2	7	0.03 0.23
***MMP7***	Matrix Metalloproteinase 7 (Matrilysin, Uterine)	Gene amplification Deep deletion R2Q	11q22.2	7	0.09
***MMP8***	Matrix Metalloproteinase 8 (Neutrophil Collagenase)	Gene amplification Deep deletion P362S T30A N57D W140C E200K	11q22.2	7	0.05 0.25 0.28 0.06 0.05
***MMP9***	Matrix Metalloproteinase 9 (Gelatinase B, 92kDa Gelatinase, 92kDa Type IV Collagenase)	Gene amplification Deep deletion K356*	20q13.12	0.6	0.18
***MMP10***	Matrix Metalloproteinase 10 (Stromelysin 2)	Gene amplification Deep deletion H227Y P186S W314* F401L G208D R116S	11q22.2	7	0.73 0.35 0.07 0.06 0.04 0.46
***MMP11***	Matrix Metalloproteinase 11 (Stromelysin 3)	Gene amplification Q205=	22q11.23	1.2	0.33
***MMP12***	Matrix Metalloproteinase 12 (Macrophage Elastase)	Gene amplification Deep deletion K67_splice	11q22.2	6	0.04
***MMP13***	Matrix Metalloproteinase 13 (Collagenase 3)	Gene amplification Deep deletion D429N G374R D128H P127S G248D D147N R95T M91I	11q22.2	7	0.23 0.62 0.13 0.27 0.02 0.06 0.14 0.30
***MMP14***	Matrix Metallopeptidase 14 (Membrane-Inserted)	Gene amplification Deep deletion S62L E373D F429V F467Y	14q11.2	2	0.13 0.08 0.19 0.30
***MMP15***	Matrix Metalloproteinase 15 (Membrane-Inserted)	Gene amplification Deep deletion A278V S505Rfs*11 V94L R120Qfs*2	16q21	1.6	0.31 0.22 0.08 0.40
***MMP16***	Matrix Metallopeptidase 16 (Membrane-Inserted)	Gene amplificationK339N R154H G215R R153C E518G Y268H P313H K98N	8q21.3	6	0.44 0.13 0.18 0.16 0.16 0.36 0.14 0.24
***MMP17***	Matrix Metallopeptidase 17 (Membrane-Inserted)	Gene amplificationX98_splice E228K	12q24.33	0.6	0.24 0.25
**MMP19**	Matrix Metalloproteinase-19	Gene amplification	12q13.2	0.2	-
MMP20	Matrix Metalloproteinase 20 (Enamelysin)	Gene amplification Deep deletion Q374K Q267P L282V L277P P293T L295R E434K		MMP20	Matrix Metalloproteinase 20 (Enamelysin) 0.33 0.26 0.29 0.20 0.25 0.36 0.43
**MMP21**	Matrix Metallopeptidase 21	Gene amplification I226N H501Y H392Q	10q26.2	MMP21	0.35 0.34 0.09
**MMP23B**	Matrix Metalloproteinase 23B (Femalysin)	Gene amplification Deep deletion	1p36.33	1.8	-
**MMP24**	Matrix Metalloproteinase 24 (Membrane-Inserted)	Gene amplification G367R H102L	20q11.22	2.6	0.25 0.05
**MMP25**	Membrane-Type Matrix Metalloproteinase 6	Gene amplification G187R H179R F332Sfs*35	16p13.3	1	0.16 0.28 0.11
***MMP26***	Matrix Metalloproteinase-26 (Matrilysin 2)	Gene amplificationDeep deletion H212N H37Ifs*5	11p15.4	1.2	0.13 0.36
**MMP27**	Matrix Metalloproteinase-27	Gene amplification Deep deletion D242N K493N D382N A287T H417D	11q22.2	7	0.19 0.05 0.08 0.12 0.19

**Figure 2:**
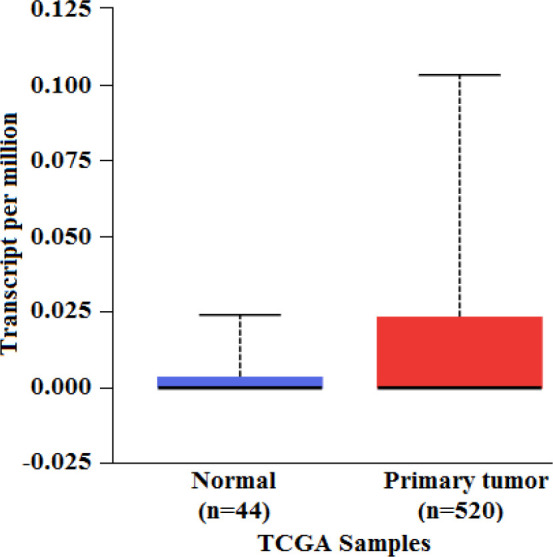
Box-Whisker plot showing relative expression of *MMP20* in primary tumor and normal tissues. The X-axis represents the type of sample and the Y-axis represents the gene expression levels presented as transcript per million. A statistically significant difference was observed between the two groups (p = 6.95 × 10^-4^).

**Figure 3:**
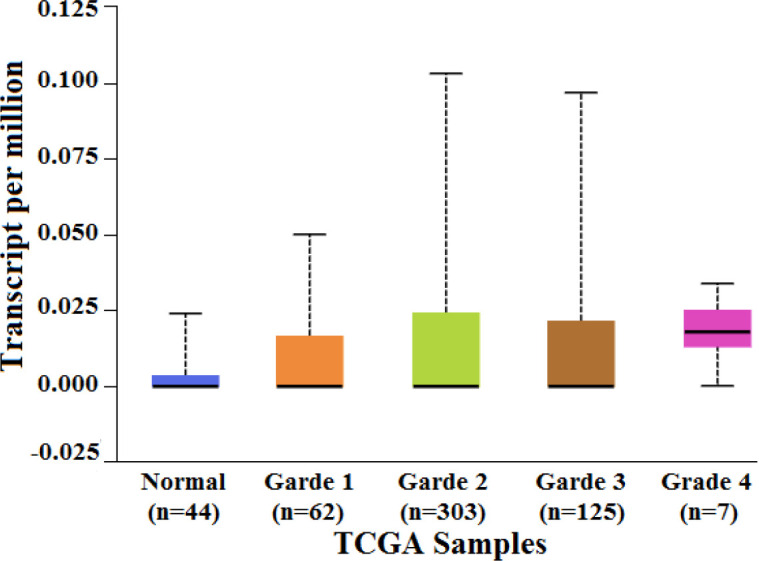
Box-Whisker plot showing relative expression profile of ​​*MMP20 *gene in different grades of HNSC. The X axis denotes the TCGA samples and Y axis denotes the transcripts per million values. The comparison of gene expression patterns between different grades of HNSC returned significant values between normal *vs* grade 2 (p = 4.84 × 10^-4^), grade 1 vs grade 4 (p = 4.08 × 10^-5^) and grade 2 *vs* grade 4 (p = 1.35 × 10^-4^).

**Figure 4:**
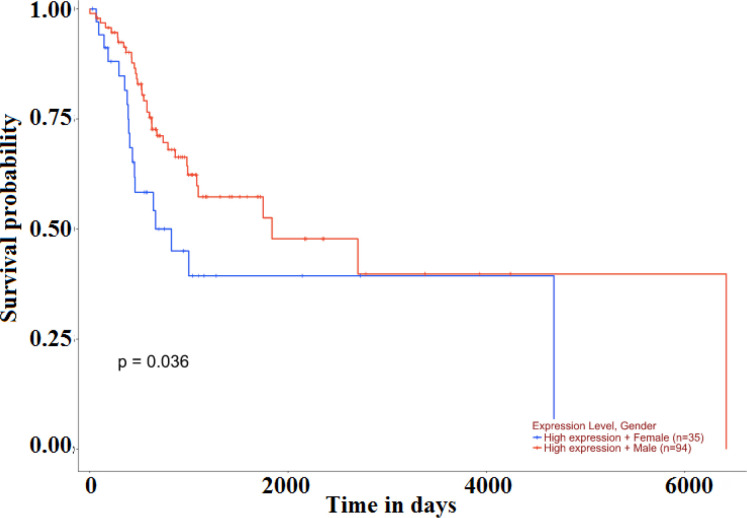
Kaplan–Meier plots showing the effect of *MMP20 *gene expression in male and female subjects with HNSC patient’s survival. The x-axis represents time in days and y-axis shows the survival probability. The blue line indicates high level expression in female patients and the red line indicates high level expression of the *MMP20 *gene in male patients. The effect of gene expression coupled with gender upon survival probability returned a significant p = 0.036. A high level expression in female subjects presented with a low survival probability when compared to male subjects

The relative gene expression showed a significant difference between normal and primary HNSC tumor tissue (p=6.95 × 10^-4^) (Fig. 2). Also, the comparison of gene expression patterns between different grades of HNSC returned significant values between normal vs grade 2 (p=4.84 × 10^-4^), grade 1 vs grade 4 (p=4.08 × 10^-5^) and grade 2 vs grade 4 (p=1.35 × 10^-4^). The results demonstrated that the level of expression of *MMP20 *increased with increasing grade of tumor (Fig. 3). The Kaplan–Meier method was used to identify the effect of *MMP20 *gene expression in male and female subjects with HNSC patient’s survival. The effect of high level expression in female patients and male patients on probability of survival returned a significant p = 0.036, wherein a high level expression in female subjects was related to low survival probability when compared to male subjects. A p value less than 0.05 is considered to be significant (Fig. 4). The results accumulated provided evidence on the putative association of *MMP20 *gene alterations with HNSC. 

## DISCUSSION

The *MMP20* gene encodes a protein enamelysin required for the normal tooth development. Apart from carryout this function, they are also involved in the breakdown of ECM which forms a part of the normal physiological process such as embryonic development, remodelling of tissues, reproduction etc., Deviations in the expression of proteins have been observed in several pathological conditions such as arthritis and metastasis. Majority of the MMPs are proproteins which are activated upon cleavage by proteinases. The MMP and its cognate the dentin sialophosphoprotein have been known to influence the process of tumorigenesis. 

A study conducted by Liu et al, investigated the expression of MMP20 in 33 cases of laryngeal squamous cell carcinoma using real time PCR assay and 73 cases employing immuno-histochemistry compared to normal epithelium. The team observed over-expression of MMP20 in LSCC when compared to the adjacent normal tissues, which implies that MMP20 could be used as a prognostic marker for lymph node metastasis [15]. The results of the present study were in agreement with the study conducted by Liu et al, wherein the dentin sialophosphoprotein (DSPP) and matrix metalloproteinase are known to react in OSCC. In line with this concept, a study was conducted by Nikitakis et al, demonstrated the effects of DSPP/MMP20 gene silencing on the expression of cancer stem cell (CSC) markers. The CSC markers viz., ABCG2 (84%) and CD44 (81%) were found to be downregulated following the double silencing. The results indicate that downregulation of MMP20/DSPP which could aid in the reduction of CSC population [16].

 The MMP20 expression was considered to be confined to dental hard tissues. A study demonstrated the association of MMP20-DSPP in human OSCC. The expression of MMP20 along with DSPP was assessed in several tumor types of breast, colon, prostate, thyroid and cervical region. The expression pattern was found to be significantly higher in all the malignant tissue types when compared to their normal counterparts. The study also documents that the level of MMP20 increased as the tumor progressed to subsequent stages, thus suggesting that this combination could be a lethal duo in the process of tumorigenesis. A very recent study by Aseervatham and Ogbureke investigated the effect of MMP20-DSPP silencing on several cancer related phenotypes such as epithelial-mesenchymal transition, cell adhesion, angiogenesis and metastasis. The team reported that the expression of certain crucial protein markers viz., *MMP2, MMP9*, integrins, *VEGF *was significantly decreased with a concomitant increase in the expression of E-cadherin. Thus these results indicate that the tumorigenic effect of MMP20/DSPP is mediated by the up-regulation of genes potentially involved in steps leading to tumor development, progression and invasion [17, 18].

Mutations in the genes encoding MMPs have shown to exert deleterious effects. Studies pertaining to the association between matrix metallo-proteinases polymorphisms with the risk of oral cancer have provided insights into the importance of human genetic variations upon susceptibility to a genetic disorder. The meta-analysis conducted by Zhang et al, identified the promoter polymorphism in *MMP2* gene (-1306 C>T) to be significantly associated with oral and nasopharyngeal cancer. Also, a subgroup analysis based on ethnicity and tumor site identified the polymorphism of the promoter element -1171 5A>6A to be associated with HNSC risk in European population [19]. A meta-analysis on the association of risk of oral cancer with MMP polymorphisms had identified *MMP-1 *(-1607) as a significant genetic marker related to oral cancer risk in Asian and protectiveness in European groups [20]. A similar study by de Matos et al, revealed that the SNP rs2252070 in *MMP-13 *gene conferred protection against oral and oropharyngeal SCC. Furthermore, the cumulative effects of *IL-8 *(rs4073), *MMP-13 *(rs2252070) and two other polymorphisms of *MMP-1 *(rs2071230, rs470558) in connection with environmental carcinogens, tobacco and alcohol was related to increased risk of oral and oropharyngeal SCC development [21]. A genotyping study conducted by Lin et al., identified the combined effect of environmental factors and polymorphism of the MMP-11 gene to be associated with the susceptibility of OSCC. They observed that patients heterozygous or homozygous for the C allele of *MMP-11 *(rs738792) polymorphism presented with an increased incidence of lymph node metastasis in comparison to patients homozygous for T allele [22]. Tu et al., demonstrated that polymorphism of MMP-9 C>T although was not associated directly with OSCC or OSF, stratification of case subjects based on median age, found a strong association with the risk of OSCC in young patients (p = 0.0029). Thus, it was concluded that the impact of aging and habits such as areca nut chewing are the influencing factors of OSCC risk [23]. Numerous reports gathered through experimental approaches and meta-analysis have provided substantial evidence on the association of genetic alterations and variations with HNSCC [24]. 
